# Multi-Solvent Graph Neural Network for Reduction Potential Prediction Across the Chemical Space

**DOI:** 10.1021/acs.jcim.5c01450

**Published:** 2026-01-12

**Authors:** Rostislav Fedorov, Anastasiia Nihei, Ganna Gryn’ova

**Affiliations:** † 40092 Heidelberg Institute for Theoretical Studies (HITS gGmbH), 69118 Heidelberg, Germany; ‡ Faculty of Engineering Sciences, Heidelberg University, 69117 Heidelberg, Germany; § School of Chemistry, 1724 University of Birmingham, B15 2TT Birmingham, U.K.

## Abstract

Reduction potentials of redox-active molecules and materials are essential descriptors of their performance as catalysts, antioxidants, electrode materials, etc. For a given species, its practical applications often span a range of solvent environments, which profoundly impact its redox properties. In this work, we present a message passing graph neural network architecture with a Set Transformer readout trained on *ca*. 20,000 reduction potentials of chemically diverse closed- and open-shell organic redox-active molecules (the “ReSolved” data set), computed using a rigorously benchmarked density functional theory procedure. The predictor model affords high accuracy with mean absolute errors of *ca*. 0.2 eV and is uniquely able to generalize to previously unseen solvents. We couple this architecture with an evolutionary algorithm to inverse-design synthetically accessible candidate molecules with target reduction potentials for several battery-related practical applications.

## Graphical abstract

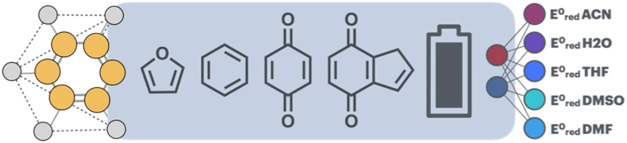

## 1 Introduction

Reduction potential is a key property of redox-active molecules and materials, determining their energetic tendency to accept an electron and critical to a plethora of their practical applications, such as electrochemical energy storage,^1^ photo- and electrocatalysis,^2,3^ and medical imaging.^4,5^ Diverse computational approaches exist to accurately (within the chemical accuracy) estimate the reduction potential of a molecule, *E*_red_, typically requiring *ab initio* computations on several closed- and open-shell neutral and charged species. Moreover, the strong dependence of the redox (reduction/oxidation) properties on the environment, *i.e*., the solvent,^6,7^ necessitates the inclusion of solvent effects in the simulations via, for example, highly parametrized continuum solvent models.^8,9^ Overall, the high computational cost and complexity of these simulations prohibits broader exploration of the vast chemical space of promising species in a multitude of practically relevant solvents. To address this challenge, data-driven approaches instead utilize relatively large data sets of molecules and their experimentally measured or computationally estimated redox potentials to elucidate the underlying structure–property patterns and produce speedy yet reasonably reliable predictions. In our 2023 perspective,^10^ we discussed representative efforts employing kernel-based machine learning (ML) methods, deep learning, and Δ-ML approaches to predicting molecular redox potentials and designing bespoke redox-active molecules. For example, Carvalho et al.^11^ developed a high-throughput screening workflow, in which a neural network architecture was trained on a data set of *ca*. 27,000 organic molecules (represented with SMILES) and their density functional theory (DFT) computed redox potentials and then applied to screen 20 million molecules from the GDB17 data set.^12^ In this manner, 459 molecules were identified as promising organic electrode materials for lithium-ion battery cathodes. Targeting aqueous redox flow batteries, Shree Sowndarya et al.^13^ developed a reinforcement learning agent based on two graph neural networks to identify organic free radicals with optimal redox properties, stability, and synthesizability. Very recently, Si et al.^14^ developed a chemical language model-based deep learning method, TransChem, for redox potential prediction using several external data sets; while the model can generalize across these data sets, predictions are still limited to one chosen solvent at a time. In fact, an overwhelming majority of studies tackling redox potential predictions with machine learning to date cater either to a single solvent, most commonly water (for biological applications)^15^ or acetonitrile (for energy conversion systems),^12^ or no solvent at all.^16^ Yet, many redox-active molecules find practical uses across a range of solvents. For example, nitroxide (nitroxyl, aminoxyl) radicals are used in water as biomedical imaging agents and antioxidants,^17^ and in diverse inorganic solvents (acetonitrile, toluene, dimethylformamide, tetrahydrofuran, etc.) as control agents in free-radical polymerizations,^18^ stabilizing additives in plastics,^19^ and redox mediators in solar cells.^20^ This prompts the need for models that can predict redox potentials not only accurately and rapidly, but also in diverse solvents. Very recently, Sharma et al.^21^ used a simple linear fit of computed energies of the highest occupied molecular orbital (HOMO) to experimentally measured aqueous oxidation potentials to predict the latter for a handful of molecules in acetonitrile. While encouraging, these results are limited both to a fraction of the chemical space and to the arguably easier-to-model oxidation properties.

Considering the solvation effects (solubility) alone, existing approaches can be broadly divided into (i) those that incorporate solvation into the training data and do not feature any additional solvent-specific representation, and (ii) models that introduce a solvent-specific descriptor and can hypothetically perform a zero-shot solvent generalization. An example of the first type is a set of machine learning models for predicting the solubility of organic molecules in water and organic solvents, developed by Boobier et al.^22^ While these single-solvent models tend to approach experimental errors, they rely on DFT to generate the required molecular descriptors and do not offer transferability across solvents. In the models of the second type, the solvent-specific descriptor can be introduced as a learned representation from an additional graph,^23–25^ or via SMILES input.^26,27^ The learned representation approach, although very expressive in theory, can be hampered by the limited diversity of solvents in the training data. More compact physics-based descriptors tend to afford better generalizability in a limited variety of solvents regime, but struggle if the physical descriptors of distinct solvents have similar values. For example, a polarity-based descriptor does not discriminate between hexane and heptane, which have almost identical polarity metrics.^25^

In this study, we address the “single solvent” limitation of existing predictive architectures for reduction potentials by constructing and training a graph neural network (GNN) capable of generalizing across solvents. Our model incorporates solvent-specific features, such as dielectric constant and refractive index, and is trained on redox potentials of closed- and open-shell redox active molecules in five solvents, computed using a rigorously benchmarked protocol based on thermodynamic cycles and DFT. Finally, we combine this predictor model with an evolutionary algorithm to design new, synthesizable candidate molecules with reduction potentials tailored to diverse practical applications.

## 2 Methods

### 2.1 Benchmarking on Experimental Data

To validate the *in silico* protocol for computing the reduction potentials, we used literature-reported experimental reduction potentials in acetonitrile for five subsets of structurally diverse organic molecules (156 molecules in total): (a) common small organic molecules,^28^ (b) polycyclic aromatic hydrocarbons (PAHs),^29^ (c) para-quinone derivatives,^30^ (d) quinones,^31^ and (e) molecules with “flexible” π-systems, PAHs, and heterocyclic amines.^9^ This selection includes “overlaps”, *i.e*., measurements on the same molecules: subset (e) includes 4 molecules from subset (a), 13 molecules from subset (b), and 9 molecules from subset (d), while subsets (c) and (d) share 6 molecules (see Supporting Information). All literature-sourced values were adjusted to the standard hydrogen electrode (SHE) as a reference electrode.

One-electron reduction potentials of the molecules in the five literature-sourced subsets at 25 °C in acetonitrile were computed as^32^

1 $$E_{red}\left(\mathrm{solv}\right)=\frac{-\Delta G_{red}\left(\mathrm{solv}\right)}{F}-E_{\mathrm{REF}}$$

where (solv) denotes the chosen solvent, Δ*G*_red_(solv) is the Gibbs free energy of reduction, obtained via the thermodynamic cycle in Figure 1, *F* is Faraday’s constant (96485.3383 C mol^–1^), and *E*_REF_ is the electrode potential of the reference electrode, SHE, equal to −4.48 V (Fermi–Dirac statistics) in acetonitrile. In calculating the Δ*G*_red_(solv), we used the Gibbs free energy of the gas-phase electron under electron convention (Fermi–Dirac statistics), equal to −3.632 kJ mol^–1^.^33^ Gas-phase Gibbs free energies of the parent and reduced molecules were computed in conjunction with their optimized geometries and frequencies at several levels of theory: B3LYP-D3/6–311G­(d,p), PBE0-D3/def2-TZVPD, and M06–2X/def2-TZVPD. In all computations, wave function stability checks were performed. For all open-shell compounds, the expectation value of the spin-squared operator was assessed, and species with ⟨S2⟩ above 10% of the fully single-reference expectation value were excluded. All species with any negative frequencies were removed from the benchmark data set. Solvation free energies in acetonitrile were computed using continuum solvent models, *i.e*., the Conductor-like Polarizable Continuum Model (CPCM) in conjunction with B3LYP and PBE0, and Solvation Model Density (SMD) at the M06–2X/cc-pVTZ level, using van der Waals atomic radii model. All computations were performed using ORCA 5.0.3 and Gaussian 16 codes.^34–36^ Of the tested levels of theory, SMD/M06–2X/def2-TZVPD afforded the best agreement with experimental values, as reflected by a mean absolute error (MAE) of just 0.11 eV, an accuracy better than the reported experimental precision (Figure 2, see Supporting Information for the full details of the methods benchmark). Correspondingly, this method was chosen for all subsequent computations.

**Figure 1:**
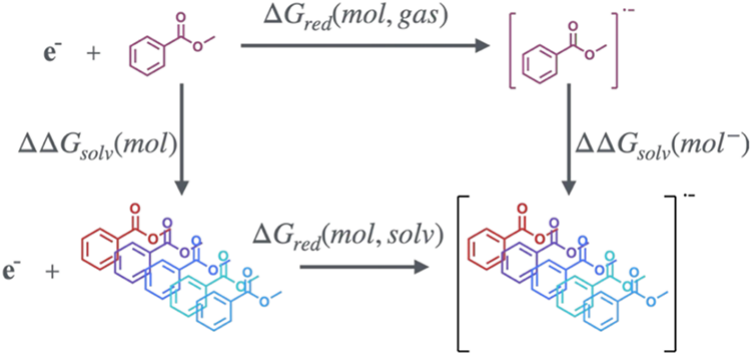
Exemplary thermodynamic cycle used to compute the reduction potential of molecule *mol* in solvent *solv*. Colors denote distinct solvents.

**Figure 2:**
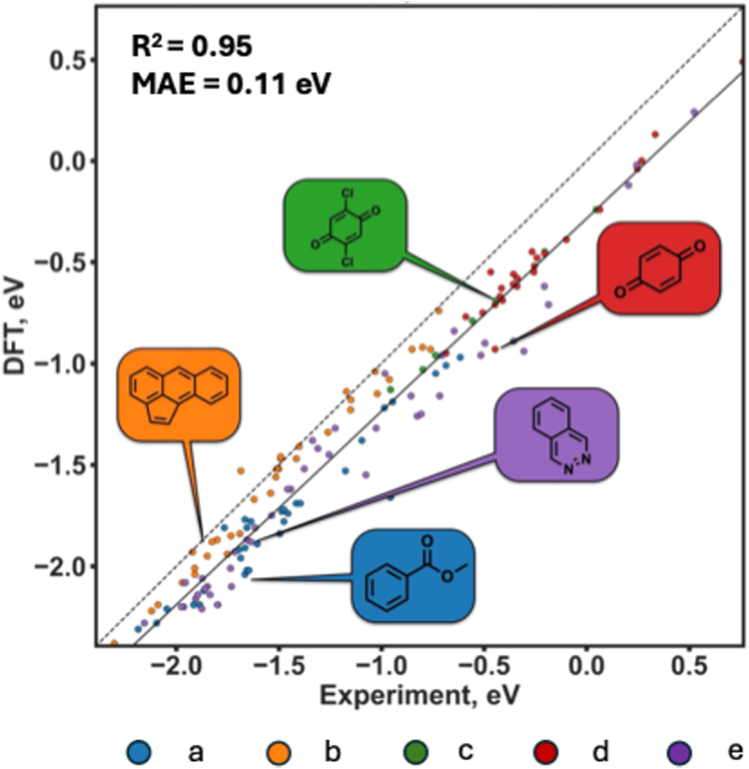
Literature-sourced experimental (*x*-axis) and M06–2X computed (*y*-axis) reduction potentials in acetonitrile at 25 °C for five subsets of molecules (a–e). Insets show representative molecules from each subset. Solid black line is the best linear fit, dashed black line is the *x = y* line.

### 2.2 ReSolved Data Set

A dedicated data set called “ReSolved” (Reduction in Solvents) was constructed to train and test the machine learning models. This data set includes molecules from existing sets, namely, the “OMEAD” (Organic Materials for Energy Applications Database)^11^ data set of molecules for energy-related applications, and the REDOX data set^37^ containing organic radicals (nitroxyl, phenoxyl, and galvinoxyl), carbonyl compounds (quinones, carboxylates, and phenazine-derived radicals), and cyanides. Reduction potentials of all species at 25 °C in five solvents – acetonitrile (ACN), water (H_2_O), tetrahydrofuran (THF), dimethyl sulfoxide (DMSO), and dimethylformamide (DMF) – as well as their electron affinities (EAs) were computed at the M06–2X/def2-TZVPD level of theory with the SMD solvent model (for *E*_red_), as described above. All molecules undergoing over 40% in bond length change upon one-electron reduction were removed as unstable, *i.e*., due to intramolecular rearrangement or decomposition upon reduction. All systems with bulk electrostatic contribution arising from a self-consistent reaction field treatment outside the −0.1 to −4.0 eV range were removed as a potentially subject to a numerical error. The resulting ReSolved data set contains DFT-computed electron affinities and one-electron reduction potentials in five solvents for 19,785 molecules (Figure 3).

**Figure 3:**
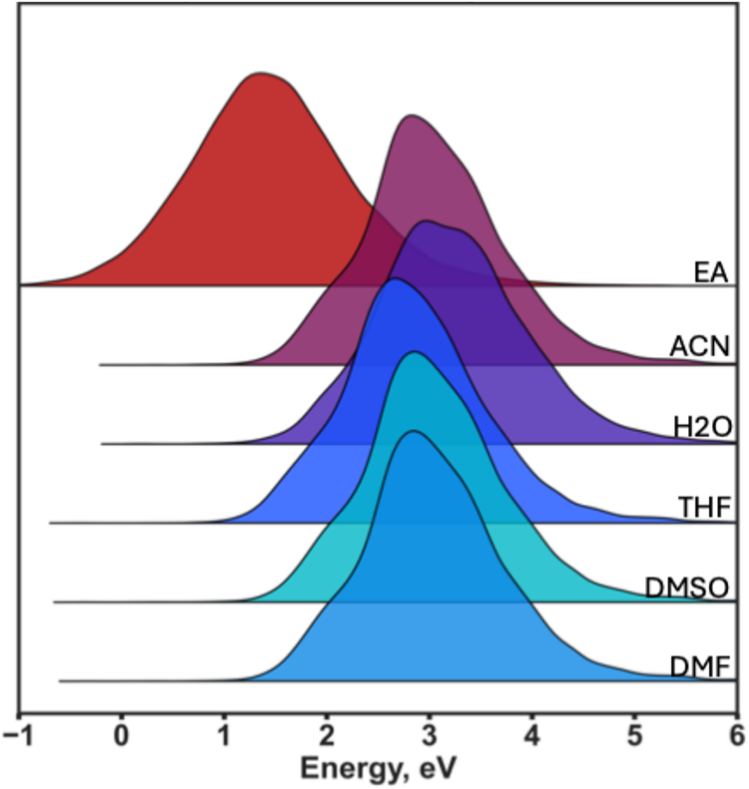
Distribution of DFT-computed electron affinities and reduction potentials in the ReSolved data set.

### 2.3 Message-Passing Neural Network

To predict reduction potentials, a combination of a message-passing graph neural network (MPNN) and a Set Transformer architecture^38^ was adopted (Figure 4, see Supporting Information for further details). First, the state of the node and edge are updated in a residual fashion. In each iteration, nodes gather information from their neighbors through a learned message function. The message each node receives depends on its own features, the features of the neighboring nodes, and the connecting edges. These messages are then added to the current node features to produce updated representations. Similarly, edge features are updated using the messages passed during this process, allowing both node and edge representations to evolve jointly over time. After the message passing is complete (six iterations), the final representations of the nodes and edges are concatenated into a single feature vector. This vector then serves as the input to the Set Transformer component, which handles the readout phase of the model. This final output from the Set Transformer serves as the global representation of the molecule, used as an input for the multilayer perceptron (MLP) predicting the solvent-independent part of the reduction potential (*i.e*., the electron affinity); the solvent description is fed into a separate MLP, predicting the solvent-dependent part of the reduction potential.

**Figure 4:**
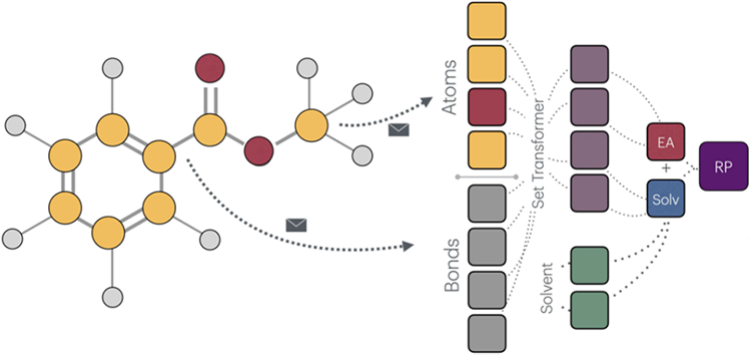
Architecture of the message-passing neural network. After several iterations of message passings, nodes and edges are concatenated into a graph vector. Set Transformer layer pools the atomic and bond features from the graph node via multihead-attention. Pooled feature vector is an input to an MLP, which predicts the EA. Concatenated feature vector including solvent description is an input to a second MLP (Solv). The outputs of the last two neurons are summed, yielding an output value – the reduction potential (RP).

Molecular graphs, used as inputs to the machine learning model, were constructed by converting the SMILES strings into molecular objects using the RDKit library^39^ and extracting the relevant features. The atom features – atom type, number of heavy atom neighbors, ring membership, aromaticity, atomic mass, van der Waals radius, covalent radius, and valence – were encoded with categorical indices for atom types and scaled values for atomic mass and radii. The bond features – bond type, conjugation status, ring membership – were encoded as categorical indices (for bond type) and as Boolean indicators (for the remaining features). The solvent is represented by its dielectric constant and refractive index (listed in the Supporting Information). They are projected into the latent space and concatenated with the graph representation vector (Figure 4).

The training process was conducted over 60 epochs, with each epoch comprising a complete pass through the training data. Mean absolute error was used as a loss function; individual losses for each output dimension (Vector of Solvents, EAs, and individual reduction potentials) were calculated and summed to give the combined loss. The epoch loss was accumulated and averaged over the number of graphs processed to obtain the training average loss for that epoch. AdamW optimizer was initialized with a learning rate of 1 × 10^–4^ and a weight decay of 1 × 10^–5^ to prevent overfitting. Throughout training, the best model was identified by comparing the validation losses across epochs. If the current epoch’s validation loss was lower than the best recorded validation loss, the model’s state was saved, and the best validation loss was updated. This process prevented the overfitting, as most of the models converged before 50 epochs. The data set (at the level of molecular graph representations) was split into training, test, and validation sets using an 80/10/10 ratio. The training data loader was configured with a batch size of 32 and shuffling was enabled. The pretrained neural network models are provided at https://github.com/grynova-ccc/ReSolved, while the ReSolved data set, including SMILES, DFT-optimized geometries and DFT-computed reduction potentials is available from https://github.com/grynova-ccc/ReSolvedDB.

### 2.4 Targeted Molecular Generation

To assess the practical utility of our predictor model in guiding the *de novo* molecular design, we coupled it with an evolutionary algorithm, EvoMol.^40^ This algorithm modifies molecules by applying predefined actions to their molecular graphs, starting from one or more seed molecules. These actions include adding or removing atoms and changing bond types, as well as more complex compound actions such as substituting atom types or moving functional groups. These operations guide the exploration of the chemical space, allowing the algorithm to generate diverse molecular structures while retaining chemical validity. Mutation is considered successful if the produced molecule lies within a certain SAscore.^41^ SAscore quantifies the ease of synthesis (synthetic accessibility) of a molecule based on rules derived from fragment contributions (a proxy for the historical synthetic knowledge) and a molecular complexity penalty. The objective function in EvoMol is crucial for guiding the evolutionary process. It is implemented as a multiobjective function with five graph neural network (GNN) models, each trained on a distinct train/test/validation split and a distinct random seed. Each model assesses the reduction potential of the generated molecules, wrapped in a linear combination of a sigmoid and a 1- sigmoid function in such a way that the scoring function would return 1 if the predicted reduction potential is in the target range, and 0 if it is outside of this predefined range.

## 3 Results and Discussion

The performance of the message-passing graph neural network trained on the ReSolved data set was assessed against the DFT-computed reduction potentials in five solvents (Figure 5A). For every molecule in the input, the output is a vector of five values, each corresponding to a distinct solvent; we refer to this model as GNN-VS (*i.e*. graph neural network with a vector of solvents). The predicted values achieve an average coefficient of determination, *R* ^2^, of 0.79 and an MAE of 0.19 eV across five solvents on the test set of 1979 molecules withheld from the training and validation sets. Interestingly, an MPNN trained exclusively on closed-shell molecules cannot generalize to stable open-shell compounds (Figure 5B). In contrast, when the model was trained on only the open-shell compounds, appreciable prediction accuracy was achieved, with an *R* ^2^ of 0.83 and an MAE of 0.13 eV (Figure 5C).

**Figure 5:**
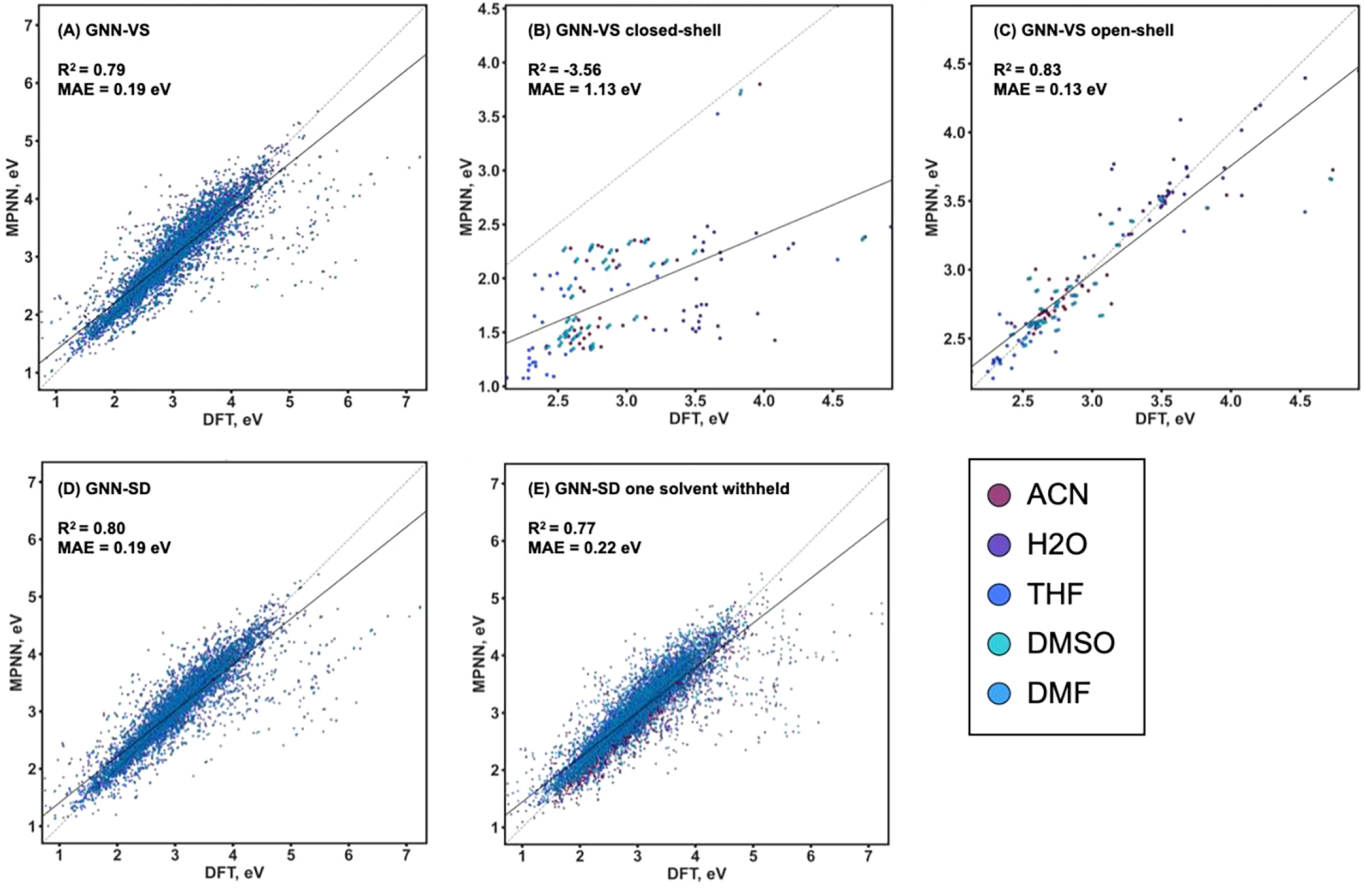
Parity plots of DFT-computed and MPNN-predicted reduction potentials for the test set of the ReSolved data set: GNN-VS model predicting a vector of reduction potentials in five solvents trained on (A) the entire data set, (B) on the closed-shell molecules only, and (C) on the open-shell species only; GNN-SD model predicting a single value of the reduction potential in a given solvent trained on (D) the entire data set in all five solvents and (E) the entire data set in four solvents, *i.e*., reduction potentials are predicted in one solvent withheld from training, and the experiment is repeated five times, once for each solvent. In all plots, solid black line is the best linear fit, dashed black line is the *x = y* line.

### 3.1 Generalizability Across Solvents

To investigate how information about the solvent affects the prediction quality, selected solvent features, *i.e*., the dielectric constant and the refractive index,^42^ were added to the readout layer of the model, which we refer to as GNN-SD (*i.e*., graph neural network with solvent description). The last hidden layer of the readout was split in two: one receiving information about the molecule and the description of the solvent, and the other receiving information about the molecule only. A linear one-dimensional layer was applied to each of these layers, their outputs were then summed, and this sum, *i.e*., the reduction potential in a given solvent, was the final output of the MPNN (Figure 4). While the prediction accuracy (Figure 5D) was largely unaffected relative to that of GNN-VS (Figure 5A), explicit separation of the solvent-independent and solvent-dependent terms in both the input and the output allowed testing the generalizability of the predictor MPNN across solvents. To this end, the GNN-SD model was retrained on the ReSolved data set, but the data in one solvent was withheld from the training set and the model’s performance was evaluated in this withheld solvent; this test was repeated five times, withholding each solvent once (Figure 5E). In the train/test/validate split on molecular graphs, once a given molecule was assigned to, e.g., the training set, all data associated with that molecule, including its solvation and redox properties, were excluded from the validation and test sets. This strategy prevents information leakage arising from shared structural features of the same molecule across solvents and therefore enables a fair assessment of the model’s ability to generalize to new molecules and new solvent environments. The overall accuracy of the model was only slightly reduced compared to training on all five solvents, to an MAE of 0.22 eV and an *R* ^2^ of 0.77. Accuracy metrics for predictions in individual (withheld) solvents (Table 1) further support the model’s robustness when generalizing to previously unseen solvents.

**Table 1:** Accuracy^a^ of Reduction Potential Predictions for the ReSolved Dataset with the GNN-VS and GNN-SD Models

	ACN		H_2_O		THF		DMSO		DMF	
MPNN model	*R* ^2^	MAE	*R* ^2^	MAE	*R* ^2^	MAE	*R* ^2^	MAE	*R* ^2^	MAE
GNN-VS	0.77	0.20	0.76	0.22	0.78	0.19	0.77	0.20	0.77	0.20
GNN-SD	0.77	0.20	0.77	0.21	0.79	0.19	0.77	0.20	0.77	0.19
GNN-SD^b^	0.75	0.23	0.73	0.25	0.75	0.24	0.79	0.20	0.76	0.20

a MAE values are in eV.

b GNN-SD experiments with data in the predicted solvent being withheld from the training to probe the model’s ability to generalize to previously unseen solvents.

### 3.2 Targeted Inverse Molecular Design

We employed EvoMol,^40^ an evolutionary algorithm for molecular graph generation, to design *de novo* synthetically accessible (evaluated via SAscore^41^) molecules with the desired reduction potentials (predicted by our MPNN, specifically, the GNN-SD architecture). Four practical applications of redox-active molecules were selected, each with a specific reduction potential window:

1. A promising strategy toward efficient and sustainable rechargeable battery materials is to combine small redox-active molecules with conducting polymers to achieve redox matching. Here, we targeted molecules that match poly­(3,4-ethylenedioxythiophene) (PEDOT), which has a potential window from −0.19 to −0.90 V vs. Fc^0^/Fc^+^ in acetonitrile.^43^
2. To redox-match another conductive polymer, polyphenylthiophene (PPT), a very narrow potential window of −1.99 to −2.24 V Fc^0^/Fc^+^ in acetonitrile^43^ must be targeted. The 0.25 eV potential range is similar to the MAE of our MPNN, representing a particularly challenging task for molecular generation.
3. For small-molecule additives in lithium-ion batteries, high reduction potentials (above 2.0 V vs. Li/Li^+^) are considered optimal.^44^ Thus, we targeted molecules with reduction potentials between 2.5 and 3.5 V vs Li/Li^+^ in DMSO.
4. Redox flow batteries (RFBs) benefit from negative anolyte potentials, as the broader voltage gap relative to the catholyte increases overall cell voltage. The recommended potential window for small-molecule anolytes spans from −0.4 to 0.2 V vs. SHE in water.^45^

In each case, the experimentally reported range of desired potentials was converted from relative (to the relevant reference electrode) to absolute and used to guide molecular evolution. Seed molecules were chosen from either known or promising candidates and evolved guided by the MPNN-predicted reduction potential, selected SAscore threshold, a maximum limit of 15 heavy atoms, and an elemental composition from C, N, O, F, S, Cl, and Br. For the candidate molecules generated by EvoMol within a preset time limit of 3 h and satisfying a stricter SAscore cutoff, we then performed geometry optimization (filtering out those molecules whose geometries did not converge with the default number of optimization steps) and computed their reduction potentials in a given solvent using the same DFT-based protocol as before. Finally, for those molecules whose DFT-computed reduction potentials fell within the target range, we evaluated the mean absolute error between the MPNN-predicted and the DFT-computed values.

The results of these tests (Figure 6) illustrate that the MPNN-EvoMol inverse design framework consistently produced potentially synthesizable candidates with the desired redox properties across diverse application targets and in diverse solvents. Notably, the predictive accuracy of the MPNN remained consistent, with mean absolute errors in the 0.08–0.26 eV range. Although certain application windows spanned only ∼0.25 eV, similar to the model’s predictive uncertainty, the successful identification of candidates within these narrow bounds suggests that the framework can effectively operate within tight property constraints, despite inherent model error.

**Figure 6:**
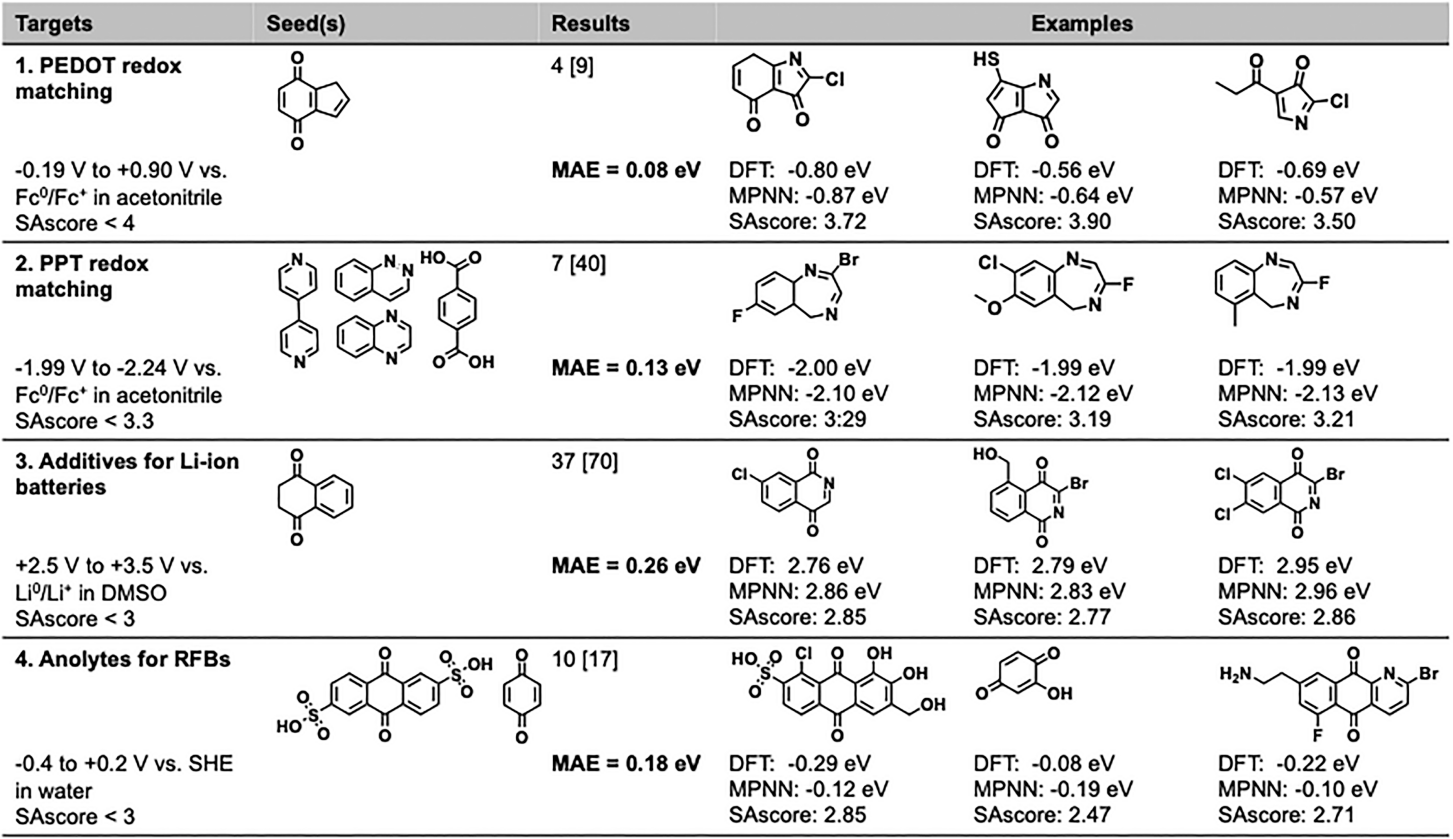
Inverse molecular design with EvoMol and pretrained MPNN. “Targets” include the practical application, the desired range of reduction potentials under relevant conditions, and the synthetic accessibility threshold. “Results” includes the number of molecules with DFT-computed potentials in the desired range, selected from the [number] of EvoMol-generated candidates, as well as the MAE between their MPNN-predicted and DFT-computed potentials. “Examples” provides three illustrative promising molecules for each application together with their DFT-computed and MPNN-predicted reduction potentials and the SAscore (see Supporting Information for a full list of generated candidates).

## 4 Conclusions

In this work, a message-passing graph neural network architecture with a Set Transformer readout comprising solvent-dependent and solvent-independent neurons was constructed and trained on a data set of redox-active organic molecules in five solvents, predicting their absolute reduction potentials with mean absolute errors of *ca*. 0.2 eV. Learning on molecular graphs and solvent features, the model was shown to effectively generalize to previously unseen solvents with negligible loss of accuracy. This approach enables fast and robust simultaneous predictions of redox properties for molecules and in solvents relevant to biological, renewable energy, and catalysis applications. Combining the pretrained neural network with the molecular evolution algorithm, we exemplified the *de novo* inverse design capabilities of this framework for four battery-related applications. For each of them, new synthetically viable candidates with optimal reduction potentials were proposed. Criteria other than the desired reduction potential and synthetic accessibility are likely relevant to various practical applications. Provided these criteria can be represented by easily computable or ML-predictable descriptors,^46^ the latter can be easily incorporated in the multiobjective function guiding the molecular evolution, as was exemplified here for the MPNN predicting the *E*_red_.

Admittedly, in this work we used a simplistic and limited metric of synthetic accessibility, the SAscore. More rigorous assessment of synthesizability can be achieved by incorporating other scores,^47^ coupling the inverse design framework with retrosynthesis ML models,^48^ and/or harvesting the candidates from existing databases of commercially available species, such as ZINC^49^ or Enamine REAL. Furthermore, at present our predictor model lacks explicit uncertainty quantification; in molecular generation with EvoMol, the uncertainty is treated implicitly using a deep ensemble,^50^ where only molecules, for which all ensemble members yield reduction potential within the targeted search window, are selected. Integrating explicit uncertainty quantification via, for example, Bayesian message-passing^51^ or Monte Carlo dropout schemes^52^ would afford more reliable screening and active-learning, as well as better control over exploration-exploitation trade-offs during the inverse design. Finally, although our model generalizes to unseen solvents, residual solvent-specific biases may remain due to data imbalance and only two solvent descriptors (dielectric constant and refractive index). The former can be addressed by retraining the model on a data set with greater solvent diversity. The latter ensures the model’s ability to generalize to unseen solvents as long as their ε and n parameters are available. This bias can be mitigated by introducing additional solvent descriptors provided they are similarly known (or easily obtainable) for a broad range of solvents.

The ReSolved data set generated in this work contains electron affinities and reduction potentials for nearly 20,000 chemically diverse closed- and open-shell redox-active organic molecules in five solvents (water, acetonitrile, tetrahydrofuran, dimethyl sulfoxide, and dimethylformamide), computed using a DFT protocol rigorously benchmarked against literature experimental data. This data set can be used not only for training machine learning models, but also for an in-depth analysis of underlying structure–property relationships through, e.g., explainable AI techniques. Finally, the MPNN model can be retrained or appended through active learning to expand its applicability to other regions of chemical space, such as redox-active inorganic species, using the relevant training sets.^53,54^

## Supplementary Material



## Data Availability

The pretrained neural network models are provided at https://github.com/grynova-ccc/ReSolved, while the ReSolved data set, including SMILES, DFT-optimized geometries and DFT-computed reduction potentials is available from https://github.com/grynova-ccc/ReSolvedDB.

## Notes

Further computational details, methods benchmarks, and full list of candidate molecules produced by the inverse design framework (PDF)

## ∥

Faculty of Chemistry and Food Chemistry, Dresden University of Technology, 01059 Dresden, Germany
