# Supplementary material for: Multi-Solvent Graph Neural Network for Reduction Potential Prediction Across the Chemical Space
Source: J Chem Inf Model. 2026 Jan 12;66(2):847–54. doi: 10.1021/acs.jcim.5c01450 (PMC12848968; doi:10.1021/acs.jcim.5c01450)
Supplement: Supplementary file 1 [file ci5c01450_si_001.pdf]

SUPPORTING INFORMATION

Multi-Solvent Graph Neural Network for Reduction Potential Prediction across the Chemical Space

Rostislav Fedorov,[1,2] Anastasiia Nihei[1†] and Ganna Gryn'ova[1,3]*

[1] *Heidelberg Institute for Theoretical Studies (HITS gGmbH), 69118 Heidelberg, Germany*

[2] *Faculty of Engineering Sciences, Heidelberg University, 69117 Heidelberg, Germany*

[3] *School of Chemistry, University of Birmingham, B15 2TT Birmingham, United Kingdom*

[†] *Present address: Faculty of Chemistry and Food Chemistry, Dresden University of Technology, 01059 Dresden, Germany.*

** Corresponding author. Email: g.grynova@bham.ac.uk*

Table of Contents

1 Methods Benchmark against Experimental Data S2

1.2 Methods benchmark S4

2 Message-Passing Neural Network S10

3 Solvent Features S13

4 Targeted Inverse Molecular Design S14

4.2 PEDOT redox matching S14

4.2 PPT redox matching S14

4.3 Additives for Li-ion batteries S15

4.4 Anolytes for redox-flow batteries........ S16

1 Methods Benchmark against Experimental Data

Dataset **a**: This dataset was sourced from Ref. 1. The original redox potential values were reported relative to the saturated calomel electrode (SCE) and were converted to standard hydrogen electrode (SHE) scale by adding +0.244 eV, the conversion factor provided in Ref. 2.

Dataset **b**: This dataset was sourced from Ref. 3, which compiled data from Ref. 4. The original values were reported relative to the ferrocene/ferrocenium couple (Fc/Fc^+) and were adjusted to SHE by adding +0.624 eV, the conversion factor provided in Ref. 2.

Dataset **c**: This dataset was sourced from Ref. 5, where redox potentials were originally reported against SCE. These values were converted to SHE by adding +0.244 eV, the conversion factor provided in Ref. 2.

Dataset **d**: This dataset was sourced from Ref. 6, where redox potentials were originally reported relative to Fc/Fc^+. These values were converted to SHE by adding +0.624 eV, the conversion factor provided in Ref. 2.

---

[1] Roth, H. G.; Romero, N. A.; Nicewicz, D. A. Experimental and Calculated Electrochemical Potentials of Common Organic Molecules for Applications to Single-Electron Redox Chemistry. *Synlett* **2016**, *27*, 714–723. DOI: 10.1055/s-0035-1561297.

[2] Pavlishchuk, V. V.; Addison, A. W. Conversion Constants for Redox Potentials Measured versus Different Reference Electrodes in Acetonitrile Solutions at 25°C. *Inorg. Chim. Acta* **2000**, *298*, 97–102. DOI: 10.1016/S0020-1693(99)00407-7.

[3] Davis, A. P.; Fry, A. J. Experimental and Computed Absolute Redox Potentials of Polycyclic Aromatic Hydrocarbons are Highly Linearly Correlated Over a Wide Range of Structures and Potentials. *J. Phys. Chem. A* **2010**, *114*, 12299–12304. DOI: 10.1021/jp106088n.

[4] (a) Koper, C.; Sarobe, M.; Jenneskens, L. W. Redox Properties of Non-Alternant Cyclopenta-Fused Polycyclic Aromatic Hydrocarbons: The Effect of Peripheral Pentagon Annelation. *Phys. Chem. Chem. Phys.* **2004**, *6*, 319–327. DOI: 10.1039/B312234D. (b) Streitwieser Jr., A.; Schwager, I. A Molecular Orbital Study of the Polarographic Reduction in Dimethylformamide of Unsubstituted and Methyl-Substituted Aromatic Hydrocarbons. *J. Phys. Chem.* **1962**, *66*, 2316–2320. DOI: 10.1021/j100818a008. (c) Parker, V. D. Energetics of Electrode Reactions. II. The Relationship Between Redox Potentials, Ionization Potentials, Electron Affinities, and Solvation Energies of Aromatic Hydrocarbons. *J. Am. Chem. Soc.* **1976**, *98*, 98–103. DOI: 10.1021/ja00417a017. (d) Saji, T.; Aoyagui, S. Voltammetric Verification of The Hush-Blackledge Equation for the Electron Repulsion Energy. *J. Electroanal. Chem. Interf. Electrochem.* **1983**, *144*, 143–152. DOI: 10.1016/S0022-0728(83)80152-1. (e) Kubota, T.; Kano, K.; Uno, B.; Konse, T. Energetics of the Sequential Electroreduction and Electrooxidation Steps of Benzenoid Hydrocarbons. *Bull. Chem. Soc. Jpn.* 1987, *60*, 3865–3877. DOI: 10.1246/bcsj.60.3865. (f) Pysh, E. S.; Yang, N. C. Polarographic Oxidation Potentials of Aromatic Compounds. *J. Am. Chem. Soc.* **1963**, *85*, 2124–2130. DOI: 10.1021/ja00897a019.

[5] Sasaki, K.; Kashimura, T.; Ohura, M.; Ohsaki, Y.; Ohta, N. Solvent Effect in the Electrochemical Reduction of p-Quinones in Several Aprotic Solvents. *J. Electrochem. Soc.* **1990**, *137*, 2437–2443. DOI: 10.1149/1.2086957.

[6] Huynh, M. T.; Anson, C. W.; Cavell, A. C.; Stahl, S. S.; Hammes-Schiffer, S. S. Quinone 1e– and 2e–/2H+ Reduction Potentials: Identification and Analysis of Deviations from Systematic Scaling Relationships. *J. Am. Chem. Soc.* **2016**, *138*, 15903–15910. DOI: 10.1021/jacs.6b05797.

Dataset **e**: This dataset, sourced from Ref. 7, represents a combination of data from fourteen literature sources; some of the data was re-measured by the authors of Ref. 7. The redox potentials were originally reported against SCE and converted to SHE by adding +0.244 eV, the conversion factor provided in Ref. 2.

The inclusion of multiple datasets from varied literature sources and experimental setups allows for cross-verification of the data, enabling identification and exclusion of the outliers.

Table S1. Overlapping molecules between the literature-sourced experimental datasets.

SMILES	ID	Reduction potential, eV				
		As reported in original sources		Converted to an SHE scale		Δ
Dataset		**e**	**d**	**e**	**d**	
O=C(C(C(C)=C1C)C(C)=C(C)C1=O	d(122)	-0.8	-1.213	-0.556	-0.589	-0.033
O=C(C(C(C)=C1)C(C)=C(C)C1=O	d(120)	-0.75	-1.131	-0.506	-0.507	-0.001
O=C(C(C=C1C)C=C(C)C1=O	d(118)	-0.63	-1.034	-0.386	-0.41	-0.024
O=C(C(C=C1)C=C(C)C1=O	d(115)	-0.58	-0.963	-0.336	-0.339	-0.003
O=C(C=C1)C=CC1=O	d(113)	-0.47	-0.881	-0.226	-0.257	-0.031
O=C(C=C1)C=C(Cl)C1=O	d(127)	-0.34	-0.723	-0.096	-0.099	-0.003
O=C(C(F)=C1F)C(F)=C(F)C1=O	d(124)	0.02	-0.377	0.264	0.247	-0.017
O=C(C(Cl)=C1Cl)C(Cl)=C(Cl)C1=O	d(126)	0.05	-0.356	0.294	0.268	-0.026
O=C(C(C#N)=C1C#N)C(Cl)=C(Cl)C1=O	d(123)	0.59	0.137	0.834	0.761	-0.073
					average	*-0.023*
Dataset		**e**	**b**	**e**	**b**	
C1CCC2CCCCC2C1	b(88)	-2.66	-2.986	-2.416	-2.362	-0.054
C1=CC2=C3C(=C1)C=CC3=CC=C2	b(56)	-1.8	-2.125	-1.556	-1.501	-0.055
C1=CC=C2C=C3C=CC=CC3=CC2=C1	b(69)	-1.06	-1.385	-0.816	-0.761	-0.055
C1CCC2CC3CCCCC3CC2C1	b(59)	-2.1	-2.425	-1.856	-1.801	-0.055
C1CCC2C(C1)CCC3CCCCC23	b(92)	-2.62	-2.937	-2.376	-2.313	-0.063
C1=CC=C2C3=C4C(=CC=CC4=CC2=C1)C=C3	b(55)	-1.47	-1.795	-1.226	-1.171	-0.055
C1=CC=C2C(=C1)C=C3C=CC4=C3C2=CC=C4	b(57)	-1.81	-2.125	-1.566	-1.501	-0.065
C1=CC=C2C(=C1)C3=CC=CC4=C3C2=CC=C4	b(86)	-1.92	-2.245	-1.676	-1.621	-0.055
C1CC2CCC3CCCC4CCC(C1)C2C34	b(94)	-2.22	-2.534	-1.976	-1.91	-0.066
C1CC2CCC3CCC4CCCC5C(C1)C2C3C45	b(62)	-1.84	-2.165	-1.596	-1.541	-0.055
C1CC2CCC3CCC4CCC5CCC1C1C2C3C4C15	b(76)	-1.99	-2.315	-1.746	-1.691	-0.055

[7] Lynch, E. J.; Speelman, A. L.; Curry, B. A.; Murillo, C. S.; Gillmore, J. G. Expanding and Testing a Computational Method for Predicting the Ground State Reduction Potentials of Organic Molecules on the Basis of Empirical Correlation to Experiment. *J. Org. Chem.* **2012**, *77*, 6423–6430. DOI: 10.1021/jo300853k.

C1CC2CCCC3C4CCCC5CCCC(C(C1)C23)C45	b(91)	-1.81	-2.139	-1.566	-1.515	-0.051
					average	*-0.057*
Dataset		**e**	**a**	**e**	**a**	
CC(=O)C1CCCCC1	a(11)	-2.1	-2.11	-1.856	-1.866	0.01
CC(=O)C1CCC(C)CC1	a(8)	-2.2	-2.16	-1.956	-1.916	-0.04
COC(=O)C1CCCCC1	a(3)	-2.22	-2.34	-1.976	-2.096	0.12
N#CC1CCC(CC1)C#N	a (34)	-1.6	-1.64	-1.356	-1.396	0.04
					average	*0.033*
Dataset		**e**	**c**	**e**	**c**	
O=C1C(Cl)=CC(C(Cl)=C1)=O	c(97)	-0.18	-0.197	0.064	0.047	0.017
Dataset		**c**	**d**	**c**	**d**	
O=C(C=C1)C=CC1=O	d(113)	-0.522	-0.881	-0.278	-0.257	-0.021
O=C(C=C1)C=C(C)C1=O	d(115)	-0.609	-0.963	-0.365	-0.339	-0.026
O=C(C(C#N)=C1C#N)C(Cl)=C(Cl)C1=O	d(123)	0.502	0.137	0.746	0.761	-0.015
O=C(C(Cl)=C1Cl)C(Cl)=C(Cl)C1=O	d(126)	-0.005	-0.356	0.239	0.268	-0.029
O=C1C=CC(C2=C1C=CC=C2)=O	d(128)	-0.706	-1.07	-0.462	-0.446	-0.016
O=C(C1=C2C=CC=C1)C3=CC=CC=C3C2=O	d(129)	-0.952	-1.31	-0.708	-0.686	-0.022
					average	*-0.022*

1.2 Methods benchmark

Table S2. Results of the methods benchmark against experimental reduction potentials in acetonitrile at 25 °C.

Level of theory	R_2	MAE, eV	Number of molecules
SMD/M06-2X/cc-pVTZ//M06-2X/def2-TZVPD	0.95	0.11	149
CPCM/PBE0-D3/def2-TZVPD	0.91	0.13	156
CPCM/B3LYP-D3/6-311G(d,p)	0.93	0.11	148

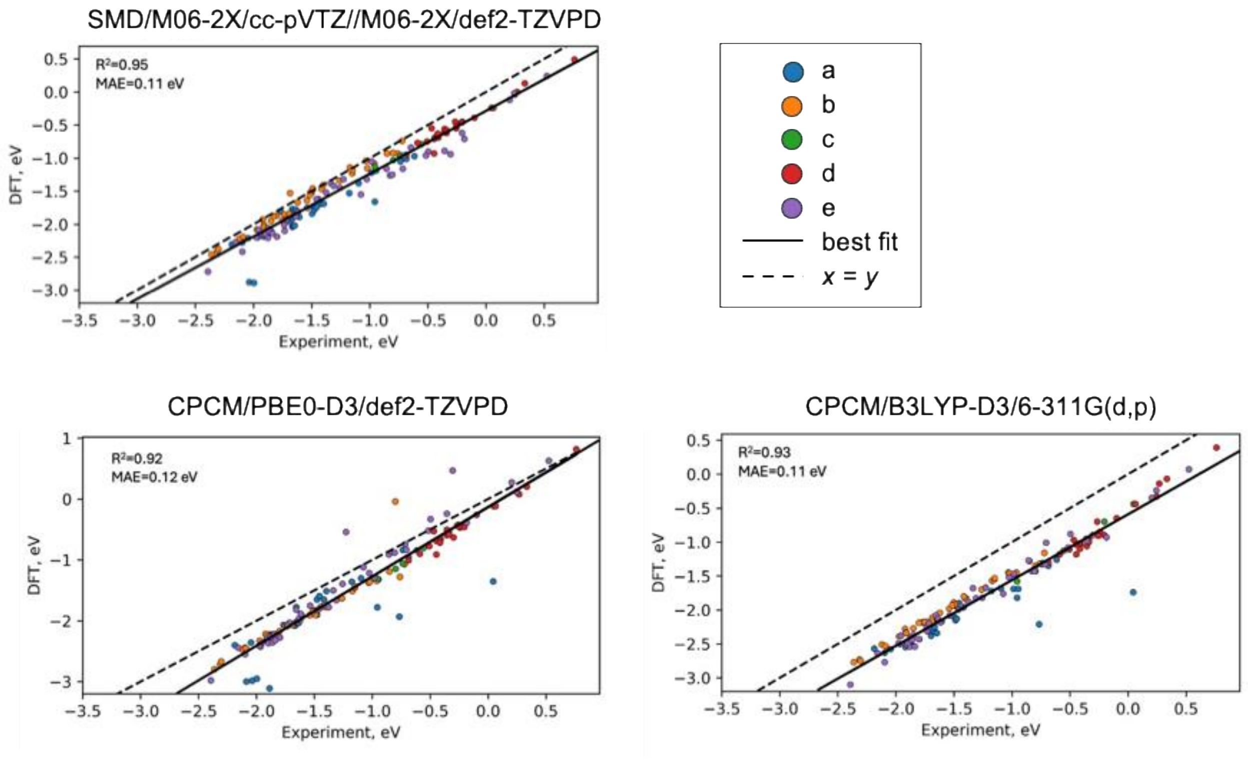

Figure S1. Literature-sourced experimental (x-axis) and DFT-computed (y-axis) reduction potentials in acetonitrile at 25°C for five literature-sourced datasets.

Table S3. Reduction potentials for the molecules in datasets **a-e**, sourced from literature (as reported in original papers and adjusted relative to SHE) and computed at the SMD/M06-2X/cc-pVTZ//M06-2X/def2-TZVPD, CPCM/PBE0-D3/def2-TZVPD, and CPCM/B3LYP-D3/6-311G(d,p) levels of theory in acetonitrile at 25 °C (relative to SHE). Empty cells denote species with convergence issues or undergoing significant structural change (bond elongation) upon reduction).

ID		SMILES	Reduction potential, eV				
			Experiment		Computed		
			Original	*vs.* SHE	M06-2X	PBE0	B3LYP
a	2	N#CC1CCCCC1	-2.430	-2.186	-2.314	-2.401	-2.571
a	3	COC(=O)C1CCCCC1	-2.340	-2.096	-2.283	-2.442	-2.626
a	4	O=C1CCCCC1	-2.330	-2.086		-2.999	-3.540
a	5	OC(=O)C1CCCCC1	-2.290	-2.046	-2.207	-2.358	-2.577
a	6	O=CC1CCCCC1	-2.280	-2.036	-2.881	-2.985	-3.394
a	7	CC(C)CC=O	-2.240	-1.996	-2.886	-2.952	-3.419
a	8	CC(=O)C1CCC(C)CC1	-2.160	-1.916	-2.187	-2.326	-2.550
a	9	O=C1CCCO1	-2.130	-1.886		-3.111	
a	10	CC(=O)C1CCC(F)CC1	-2.130	-1.886	-2.191	-2.226	-2.495
a	11	CC(=O)C1CCCCC1	-2.110	-1.866	-2.120	-2.225	-2.458

a	12	CC1=CC=C(C=C1)/N=C/C1=CC=CC=C1	-2.010	-1.766	-1.808	-2.068	-2.177
a	15	CC1CCCCC1C=O	-1.940	-1.696	-1.924	-2.062	-2.376
a	16	O=CC1CCCCC1	-1.930	-1.686	-1.962	-2.062	-2.315
a	18	C1(=CC=CC=C1)/N=C/C1=CC=CC=C1	-1.910	-1.666	-1.767	-2.037	-2.138
a	19	CC(=O)C1CCC(Cl)CC1	-1.910	-1.666	-2.041	-2.080	-2.321
a	20	CC(=O)C1CCC(CC1)C2CCCCC2	-1.910	-1.666	-1.915	-2.036	-2.249
a	21	FC1=CC=C(N=CC2=CC=CC=C2)C=C1	-1.900	-1.656	-1.808	-2.024	-2.148
a	22	N#CC1CCCC(C1)C#N	-1.900	-1.656	-2.018	-1.776	-2.118
a	23	CC(=O)C1CCC(Br)CC1	-1.890	-1.646	-2.022	-2.041	-2.335
a	24	COC=1C=C(C=CC1)/N=C/C1=CC=CC=C1	-1.880	-1.636	-1.779	-2.037	-2.137
a	25	ClC1CCC(C=O)CC1	-1.850	-1.606	-1.888	-1.923	-2.173
a	28	CC(=O)C1CCC(CC1)C(F)(F)F	-1.740	-1.496	-1.839	-1.828	-2.101
a	29	O=CC1CCC2CCCCC2C1	-1.730	-1.486	-1.731	-1.844	-2.149
a	30	O=CC1CCC(CC1)C2CCCCC2	-1.720	-1.476	-1.780	-1.901	-2.128
a	31	COC(=O)C1CCC(CC1)C#N	-1.720	-1.476	-1.716	-1.651	-1.950
a	32	N#CC1CCCCC1C#N	-1.700	-1.456	-1.736	-1.594	-1.900
a	33	FC(F)(F)C1CCC(C=O)CC1	-1.660	-1.416	-1.694	-1.654	-1.959
a	34	N#CC1CCC(CC1)C#N	-1.640	-1.396	-1.694	-1.512	-1.832
a	37	O=CC1CCC(CC1)C#N	-1.420	-1.176	-1.530	-1.359	-1.723
a	39	O=C1OC(=O)C2CCCCC12	-1.340	-1.096	-1.381	-1.302	-1.675
a	40	[O-][N+](=O)C1CCC(F)CC1	-1.230	-0.986	-1.218	-1.304	-1.690
a	41	ClC(=O)C1CCCCC1	-1.200	-0.956	-1.664	-1.778	-1.817
a	42	[O-][N+](=O)C1CCCCC1	-1.190	-0.946	-1.188	-1.319	-1.686
a	45	O=C(OC(=O)C1CCCCC1)C2CCCCC2	-1.010	-0.766		-1.932	-2.207
a	46	O=C1OC(=O)C=C1	-0.980	-0.736	-1.048	-0.838	-1.307
a	47	CC(=O)C1CCC(CC1)[N+]([O-])=O	-0.930	-0.686	-1.011	-0.956	-1.352
a	48	[O-][N+](=O)C1CCC(C=O)CC1	-0.860	-0.616	-0.970	-0.845	-1.256
a	54	FC(F)(F)C(=O)OC(=O)C(F)(F)F	-0.200	0.044		-1.353	-1.739
b	55	C1CCC2C(C1)CC3CCCC4C=CC2C34	-1.795	-1.171	-1.139	-1.512	-1.569
b	56	C1CC2CCCC3C=CC(C1)C23	-2.125	-1.501	-1.519	-1.881	-1.888
b	57	C1CCC2C(C1)CC3C=CC4CCCC2C34	-2.135	-1.511	-1.517	-1.883	-1.929
b	58	C1=CC2CCC3C=CC4CCC(C=C1)C2C34	-2.115	-1.491	-1.467	-1.870	-1.927
b	59	C1CCC2CC3CCCCC3CC2C1	-2.425	-1.801	-1.871	-2.170	-2.193
b	60	C1CCC2CCCC2CC1	-2.115	-1.491	-1.460	-1.905	-1.922
b	61	C1CCC2CC3C(CCC4CCCCC34)CC2C1	-2.473	-1.849	-1.948	-2.214	-2.271
b	62	C1CC2CCC3CCC4CCCC5C(C1)C2C3C45	-2.165	-1.541	-1.636	-1.908	-1.978
b	63	C1CC2CCC3CCC4CCC5CCCC6C(C1)C2C3C4C56	-2.358	-1.734	-1.854	-2.085	-2.172
b	64	C1CCC2C(C1)CC3CCC4CCCC5CCC2C3C45	-2.309	-1.685	-1.535	-2.043	-2.112
b	65	C1CCC2C(C1)C3CCCC4CCC5CCCC2C5C34	-2.595	-1.971	-2.081	-2.326	-2.389
b	66	C1CCC2CC3C4CCCCC4C5CCCCC5C3CC2C1	-2.545	-1.921	-1.927	-2.215	-2.280
b	67	C1CCC2C(C1)CCC3C4CCCCC4CCC23	-2.745	-2.121	-2.225	-2.472	-2.510
b	68	C1CC2CCC3CCC4CCC5CCC6CCC1C7C2C3C4C5C67	-2.526	-1.902		-2.277	-2.374
b	69	C1=CC=2C=CC3=C4C(=CC=C1C24)C=C3	-1.385	-0.761	-0.933	-1.279	-1.321

b	70	C1CC2C=CC3C4C=CC5CCCC(C(C1)C23)C45	-1.655	-1.031	-1.041	-1.373	-1.449
b	71	C1CCC2C(C1)CCC3C4CCCC5C=CC(CC23)C45	-2.025	-1.401	-1.413	-1.776	-1.839
b	72	C1=CC=2C3=C1C=CC=1C4=CC=CC=C4C(=CC2)C13	-1.655	-1.031	-1.041	-1.371	-1.445
b	73	C1=CC=2C=CC=3C=4C=CC=C5C=CC=C(C6=CC=C1C2C63)C54	-1.585	-0.961	-1.079	-1.343	-1.454
b	74	C1CC2CCC3CCC4C=CC5CC(C1)C2C3C45	-1.885	-1.261	-1.344	-1.632	-1.698
b	75	C1CCC2C(C1)CCC3CC4C(CCC5CCCCC45)CC23	-2.535	-1.911	-2.011	-2.252	-2.335
b	76	C1CC2CCC3CCC4CCC5CCC1C1C2C3C4C15	-2.315	-1.691	-1.840	-2.070	-2.167
b	77	C1CCC2C(C1)CC3C4CC5CCCCC5C6CCCC(C7CCCC2C37)C46	-2.154	-1.530			-1.951
b	78	C1CCC2C(C1)CC3C4CCCCC4C5CCCC6CCC2C3C56	-2.375	-1.751	-1.940	-2.084	-2.176
b	79	C1=CC=C2C3=C(C=C4C=5C=CC=C6C5C(=CC4=C13)C=C6)C=C2	-1.775	-1.151	-1.229	-1.458	-1.552
b	80	C1=CC2CCC3C4CCC5C=CC6CCC(C7CCC1C2C37)C4C56	-1.255	-0.631			-1.116
b	81	C1=C2C=CC3=C4C(=C5C6=C(C=CC(=C1)C6=C42)C=C5)C=C3	-1.345	-0.721	-0.741	-1.073	-1.162
b	82	C1=CC=2C=CC=3C=C4C5=C(C=CC=6C=C1C2C3C65)C=C4	-1.475	-0.851	-0.933	-1.191	-1.281
b	83	C1=CC2=CC3=C4C(=CC5=CC=CC6=CC1=C2C4=C56)C=C3	-1.645	-1.021	-1.147	-1.362	-1.460
b	84	CC1C2CCCCC2C(C)C3CCCCC13	-2.450	-1.826	-1.875		-2.235
b	85	C1CC2CCC3CCC4C5CC6CCCC7CCC8CCC(C9CC(C1)C2C3C49)C5C8C76	-1.844	-1.220			-1.703
b	86	C1CCC2C(C1)C3CCCC4CCCC2C34	-2.245	-1.621	-1.671	-1.988	-2.038
b	87	C1CCC2CC3CC4CCCCC4CC3CC2C1	-2.039	-1.415	-1.469	-1.740	-1.799
b	88	C1CCC2CCCCC2C1	-2.986	-2.362	-2.457	-2.797	-2.767
b	89	C1CCC2CC3CC4CC5CCCCC5CC4CC3CC2C1	-1.773	-1.149	-1.181	-1.435	-1.529
b	91	C1CC2CCCC3C4CCCC5CCCC(C(C1)C23)C45	-2.139	-1.515	-1.559	-1.851	-1.934
b	92	C1CCC2C(C1)CCC3CCCCC23	-2.937	-2.313	-2.406	-2.719	-2.728
b	93	C1CCC2C(C1)CCC3C2CCC4C5CCCCC5CCC34	-2.714	-2.090	-2.187	-2.452	-2.528
b	94	C1CC2CCC3CCCC4CCC(C1)C2C34	-2.534	-1.910	-2.039	-2.307	-2.337
b	95	C1CCC2C(C1)C3CCCC4C5C6CCCCC6C7CCCC(C2C34)C57	-1.424	-0.800	-0.921	-0.041	-1.351
b	96	C1CCC2C(C1)C3CCCCC3C4CCCCC24	-2.927	-2.303	-2.381	-2.670	-2.752
c	97	O=C1C(Cl)=CC(C(Cl)=C1)=O	-0.197	0.0470	-0.240	-0.118	-0.440
c	98	O=C1C(C)=CC(C(C)=C1)=O	-0.684	-0.4400	-0.687	-0.666	-1.038
c	99	O=C1C(Cl)=C(Cl)C(C2=C1C=CC=C2)=O	-0.448	-0.2040	-0.448	-0.434	-0.697

c	100	O=C1C(C)=CC(C2=C1C=CC=C2)=O	-0.801	-0.5570	-0.788	-0.804	-1.135
c	101	O=C(C1=C2C=CC=C1)C3=CC(C)=CC=C3C2=O	-0.981	-0.7370	-0.961	-1.060	-1.333
c	102	O=C(C1=C2C=CC=C1)C3=CC=CC(N)=C3C2=O	-1.042	-0.7980	-1.032	-1.147	-1.430
c	103	O=C(C1=C2C=C(N)C=C1N)C3=CC=CC=C3C2=O	-1.200	-0.9560	-1.128	-1.307	-1.577
d	113	O=C(C=C1)C=CC1=O	-0.881	-0.257	-0.550	-0.426	-0.869
d	114	O=C(C=C1)C=C(C2=CC=CC=C2)C1=O	-0.866	-0.242	-0.478	-0.469	-0.851
d	115	O=C(C=C1)C=C(C)C1=O	-0.963	-0.339	-0.620	-0.546	-0.946
d	116	O=C(C=C1)C=C(C(C)(C)C)C1=O	-0.982	-0.358	-0.608	-0.592	-0.949
d	117	O=C(C=C1)C=C(OC)C1=O	-0.980	-0.356	-0.559	-0.504	-1.056
d	118	O=C(C=C1C)C=C(C)C1=O	-1.034	-0.41	-0.692	-0.662	-1.031
d	119	O=C(C=C1)C(C)=C(C)C1=O	-1.046	-0.422	-0.670	-0.652	-1.019
d	120	O=C(C(C)=C1)C(C)=C(C)C1=O	-1.131	-0.507	-0.747	-0.772	-1.107
d	121	O=C(C=C1OC)C=C(OC)C1=O	-1.092	-0.468	-0.548	-0.529	-0.967
d	122	O=C(C(C)=C1C)C(C)=C(C)C1=O	-1.213	-0.589	-0.771	-0.900	-1.175
d	123	O=C(C(C#N)=C1C#N)C(Cl)=C(Cl)C1=O	0.137	0.761	0.492	0.817	0.391
d	124	O=C(C(F)=C1F)C(F)=C(F)C1=O	-0.377	0.247	-0.041	0.121	-0.330
d	125	O=C(C(Cl)=C1)C=C(Cl)C1=O	-0.560	0.064	-0.241	-0.118	-0.439
d	126	O=C(C(Cl)=C1Cl)C(Cl)=C(Cl)C1=O	-0.356	0.268	-0.004	0.078	-0.139
d	127	O=C(C=C1)C=C(Cl)C1=O	-0.723	-0.099	-0.393	-0.260	-0.648
d	128	O=C1C=CC(C2=C1C=CC=C2)=O	-1.070	-0.446	-0.709	-0.689	-1.045
d	129	O=C(C1=C2C=CC=C1)C3=CC=CC=C3C2=O	-1.310	-0.686	-0.952	-1.007	-1.296
d	130	O=C(C1=C2C(Cl)=CC=C1)C3=CC=CC(Cl)=C3C2=O	-1.070	-0.446	-0.932	-0.912	-1.180
d	131	O=C1C(Cl)=C(Cl)C(C2=C1C=CC=C2)=O	-0.890	-0.266	-0.449	-0.434	-0.699
d	132	O=C(C(C(C)(C)C)=CC(C(C)(C)C)=C1)C1=O	-0.922	-0.298		-0.631	-0.985
d	133	O=C(C=C(C(C)(C)C)C=C1)C1=O	-0.829	-0.205	-0.462	-0.460	-0.897
d	134	O=C1C2=C(C=CC=C2)C3=CC=CC=C3C1=O	-1.039	-0.415	-0.627	-0.719	-1.099
d	135	O=C(C=CC1=C2C=CC=C1)C2=O	-0.963	-0.3385	-0.584	-0.586	-1.003
d	136	O=C1C2=C(N=CC=C2)C3=C(C=CC=N3)C1=O	-0.879	-0.255	-0.525	-0.453	-0.905
d	137	O=C(C(Cl)=C(Cl)C(Cl)=C1Cl)C1=O	-0.290	0.334	0.129	0.202	-0.067
e	138	CC1C(C)C(C#N)C(C)C(C)C1C#N	-1.900	-1.656	-1.874	-1.817	-2.076
e	139	FC1C(F)C(C#N)C(F)C(F)C1C#N	-1.100	-0.856	-1.161	-0.910	-1.271
e	140	ClC1C(Cl)C(C#N)C(Cl)C(Cl)C1C#N	-0.950	-0.706	-0.958	-0.833	-1.005
e	141	N#CC1CC(C#N)C(CC1C#N)C#N	-0.740	-0.496	-0.901	-0.331	-0.876
e	142	N#CC1CCC2CCCCC2C1	-1.980	-1.736	-2.014	-2.087	-2.249
e	143	CC1CCC(C#N)C2CCCCC12	-1.960	-1.716	-1.931	-2.033	-2.200
e	144	N#CC1CCCC2CCCCC12	-1.880	-1.636	-1.879	-1.974	-2.134
e	145	N#CC1CCC(C#N)C2CCCCC12	-1.270	-1.026	-1.318	-1.217	-1.517
e	146	N#CC1C2CCCCC2CC3CCCCC13	-1.580	-1.336	-1.377	-1.502	-1.675
e	147	N#CC1C2CCCCC2C(C3CCCCC3)C4CCCCC14	-1.470	-1.226	-1.316	-0.543	-1.674
e	148	N#CC1C2CCCCC2C(C#N)C3CCCCC13	-0.890	-0.646	-0.839		-1.149

e	149	N#CC1CCC2C(C1)C(C#N)C3CCC(CC3C2C#N)C#N	-0.450	-0.206	-0.621		-0.714
e	150	BrC1=C(Br)C(=O)C(=C(Br)C1=O)Br	0.000	0.244	-0.016	0.115	-0.238
e	151	O=C(C=C1)C(C#N)=C(C#N)C1=O	0.280	0.524	0.239	0.629	0.074
e	152	C[N+]1CCCCC1	-1.320	-1.076	-1.548	-1.119	-1.758
e	153	CC1CC[N+](C)C2CCCCC12	-1.070	-0.826	-1.264	-0.872	-1.432
e	154	CC1CCC2CCCCC2[N+]1C	-1.050	-0.806	-1.246	-0.880	-1.429
e	155	C[N+]1CCCC2CCCCC12	-0.960	-0.716	-1.156	-0.742	-1.329
e	156	C[N+]1CC(Br)CC2CCCCC12	-0.760	-0.516	-0.964	-0.519	-1.084
e	157	C[N+]1CC(CC2CCCCC12)C#N	-0.600	-0.356	-0.894	-0.240	-0.979
e	158	C[N+]1C2CCCCC2C(C3CCCCC3)C4CCCCC14	-0.550	-0.306	-0.942	0.467	-0.900
e	159	C[N+]1C2CCCCC2CC3CCCCC13	-0.430	-0.186	-0.708	-0.389	-0.934
e	160	C[N+]1C2CCCCC2C(C#N)C3CCCCC13	-0.040	0.204	-0.122	0.272	-0.354
e	161	CCOC(=O)/C=C/C(=O)OCC	-1.500	-1.256	-1.449	-1.394	-1.816
e	162	CCOC(=O)C1CCC(CC1)C(=O)OCC	-1.780	-1.536	-1.746	-1.753	-2.076
e	163	O=C(C1CCCCC1)C2CCCCC2	-1.860	-1.616	-1.809	-1.887	-2.178
e	164	CCC(=O)C1CCCCC1	-2.150	-1.906	-2.160	-2.292	-2.509
e	165	C1CCC(CC1)\C=C\C2CCCCC2	-2.200	-1.956	-2.084	-2.371	-2.384
e	166	CCOC(=O)C1CCCCC1	-2.400	-2.156	-2.280	-2.443	-2.636
e	167	C1CCC2C(C1)NNC3CCCCC23	-1.554	-1.31	-1.421	-1.662	-1.830
e	168	C1CCC2NNCCC2C1	-1.686	-1.442	-1.617	-1.769	-1.954
e	169	C1CCC2CNNCC2C1	-1.976	-1.732	-2.097	-2.080	-2.305
e	170	C1CCC2C(C1)CNC3CCCCC23	-2.118	-1.874	-2.059	-2.295	-2.396
e	171	C1CCNNC1	-2.120	-1.876	-2.209	-2.266	-2.542
e	172	C1CNCNC1	-2.340	-2.096	-2.422	-2.549	-2.769
e	173	C1CCC2C(C1)CCC3NCCCC23	-2.140	-1.896	-2.145	-2.369	-2.476
e	174	C1CCC2C(C1)CCC3CCCNC23	-2.208	-1.964	-2.198	-2.425	-2.504
e	175	C1CCC2CNCCC2C1	-2.220	-1.976	-2.205	-2.410	-2.494
e	176	C1CNC2C(C1)CCC3CCCNC23	-2.042	-1.798	-2.188	-2.274	-2.432
e	177	C12=NC=CC=C1C=CC3=C2C=CC=N3	-2.092	-1.848	-2.105	-2.239	-2.369
e	178	C12=NC=CC=C1C=CC3=C2N=CC=C3	-2.044	-1.8	-2.189	-2.276	-2.432
e	179	C1CCNCC1	-2.636	-2.392	-2.720	-2.980	-3.099
e	180	C1CCC2NCCCC2C1	-2.105	-1.861	-2.112	-2.357	-2.441
e	181	C1CCC2NC3CCCCC3CC2C1	-1.620	-1.376	-1.524	-1.776	-1.880
e	182	C1CNCCN1	-2.080	-1.836	-2.138	-2.318	-2.543
e	183	C1CCC2NC3CCCCC3NC2C1	-1.227	-0.983	-1.049	-1.293	-1.469
e	184	C1CCC2NCCNC2C1	-1.702	-1.458	-1.617	-1.805	-1.989

2 Message-Passing Neural Network

We introduce a graph $G = (V, E))$ where V is the set of nodes and E is the set of edges. First, both node and edge features are projected into the latent space.

Message passing:

$$m_v^l = \sum_{u \in N(v)} M(h_u^{l-1}, h_v^{l-1}, e_{uv}^l) \qquad \text{Eq. 1}$$

where m_v^l is the message for node v at layer l, $N(v)$ denotes the neighbors of node v, M^l is the message function for layer l, and e_{uv} represents the edge feature between nodes u and v.

Node update:

$$h_v^l = h_v^{l-1} + m_v^l \qquad \text{Eq. 2}$$

Edge update:

$$e_{uv}^l = e_{uv}^{l-1} + m_v^l \qquad \text{Eq. 3}$$

After L iterations/layers, the features are updated as h_v^L for each node v and as e_{uv}^L for each edge.

Set Transformer readout (illustrated in Figure S2): given the concatenated matrix of node and bond features $M_i = \text{concat}(\{h_v^L\}, \{e_{uv}^L\})$ for each graph i we can proceed with the readout portion:

1. Multihead attention block (MAB) processes two sets X, Y of d-dimensional vectors:

$$M\ AB(X, Y) = \text{LayerNorm}(H + rFF(H)) \qquad \text{Eq. 4}$$

with

$$H = \text{LayerNorm}(X + \text{Multihead}(X, Y, Y; \omega)) \qquad \text{Eq. 5}$$

The induced set attention block (ISAB) is

$$ISAB(X) := MAB(X, MAB(I, X)) \qquad \text{Eq. 6}$$

The idea behind using attention between every atom and edge is to incorporate the long-range interactions and the interactions between the atoms that are close to each other in the three-dimensional space, but far apart – in the graph representation.

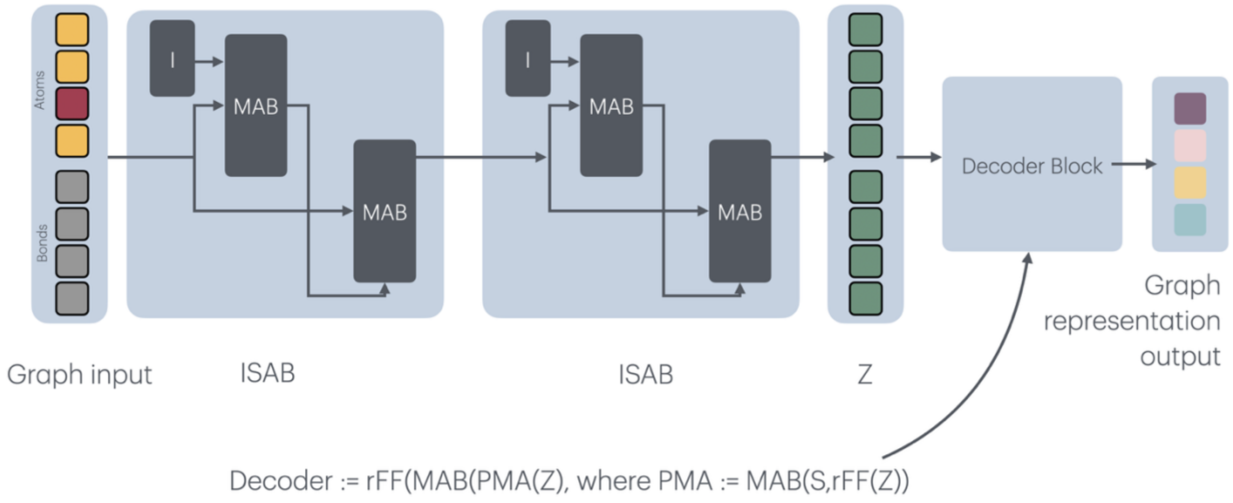

Figure S2. Schematic of a Set Transformer architecture.

2. Pooling by multihead attention (PMA) aggregates using multihead attention on a set of k learnable seed vectors S. For a feature set Z, the PMA with k seeds is:

$$PMA_k(Z) = MAB(S, rFF(Z)) \quad \text{Eq. 7}$$

The output of PMA_k contains k items. Typically, one seed vector is employed. Interactions among the k outputs are modeled using:

$$H = MAB(PMA_k(Z)) \quad \text{Eq. 8}$$

The resulting MPNN contains seven message-passing layers, the dimensions of the node and edge features are 128. The Set Transformer readout has one attention head and three seed points.

Motivation behind using the Set Transformer readout: Traditional graph-level pooling operators such as mean/sum pooling or set-invariant MLPs treat atomic embeddings independently and therefore cannot explicitly model higher-order, long-range correlations that are essential for redox chemistry (e.g., charge-transfer across conjugated fragments). The Set Transformer addresses this by replacing heuristic aggregation with a permutation-invariant multi-head attention mechanism that learns which atoms (and bonds) should attend to which others when forming the global molecular representation. In practice, we adopted the Induced Set Attention Block (ISAB) variant, where a small, trainable set of m "inducing points'' first attends to the n node-edge tokens and then re-broadcasts the context-aware summary back to them. This two-step attention reduces the

computational and memory cost from O(n^2) (full self-attention) to O(n×m). In our implementation, we set m=3, so the read-out scales linearly O(3n).

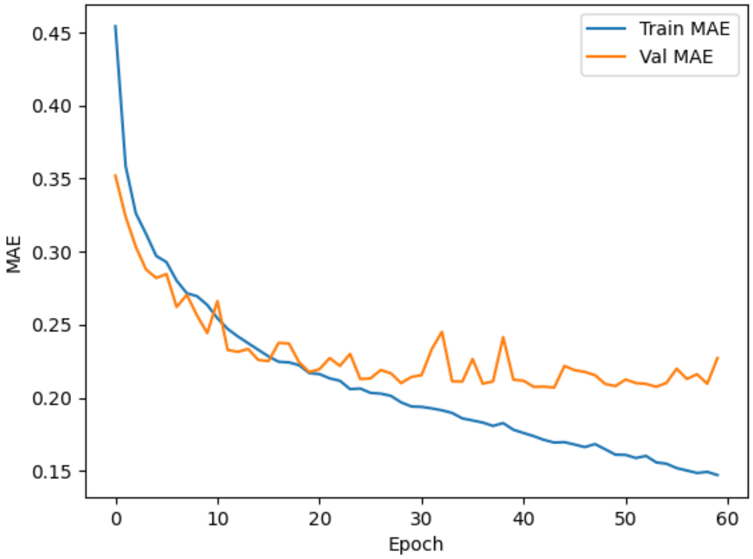

Figure S3. Learning curve of GNN-SD for all five solvents, MAE in eV. The orange curve represents loss function for the validation dataset, and the blue curve represents the loss for the training dataset.

3 Solvent Features

Table S4. Solvent features in the GNN-SD model.[8]

Solvent	Dielectric constant	Refractive index
Acetonitrile	36.6	1.344
Water	80.4	1.33
THF	7.25	1.407
DMSO	47.2	1.479
DMF	38.3	1.430

[8] CRC Handbook of Chemistry and Physics, 95th Ed.; Haynes, W.M., Ed.; CRC Press: Boca Raton, FL, 2014. DOI: 10.1201/b17118.

4 Targeted Inverse Molecular Design

4.2 PEDOT redox matching

Targeted potential window in acetonitrile: -0.19 V to -0.90 V *vs*. Fc^0/Fc^+, converted[9] to 4.21 to 4.92 V in absolute values. SAscore threshold for EvoMol = 5, filtering criteria – SAscore ≤ 4 and removal of molecules containing three- or four-membered rings.

Table S5. EvoMol-generated candidate molecules, which converged in DFT-optimisation and possess DFT-computed reduction potentials in the target window.

SMILES	SAscore	Reduction potential, eV	
		MPNN	DFT
CCCC(=O)C1=CN=C(CCl)C1=O	3.47	4.25	4.29
O=C1C=CCC2=C1C(=O)C(Cl)=N2	3.72	4.23	4.30
CCC(=O)C1=CN=C(Cl)C1=O	3.50	4.53	4.41
O=C1CNC2C(S)CC(=O)C1=2	3.90	4.46	4.54

4.2 PPT redox matching

Targeted potential window in acetonitrile: -1.99 V to -2.24 V *vs*. Fc^0/Fc^+, converted[9] to 2.87 and 3.12 V in absolute values. SAscore threshold for EvoMol = 3.9, filtering criteria – SAscore ≤ 3.3.

Table S6. EvoMol-generated candidate molecules, which converged in DFT-optimisation and possess DFT-computed reduction potentials in the target window.

SMILES	SAscore	Reduction potential, eV	
		MPNN	DFT
FC1CCC2C(C1)CN=CC(Br)=N2	3.29	3.00	3.10
CCC1CCCC2C1N=CC(F)=NC2	3.24	2.99	3.11
CC1CCCC2C1CN=C(F)C=N2	3.21	2.97	3.11
COC1CC2C(CC1Cl)N=CC(F)=NC2	3.19	2.98	3.11
FC1=NCC2CC(F)CCC2N=C1	3.21	2.98	3.11
CCC1CCCC2C1CN=C(F)C=N2	3.20	2.97	3.11
COC1CC2C(CC1Br)N=CC(F)=NC2	3.29	2.99	3.12

[9] Araujo, R. B.; Banerjee, A.; Panigrahi, P.' Yang, L.; Strømme, M.; Sjödin, M.; Araujo, C. M.; Ahujaad, R. Designing Strategies to Tune Reduction Potential of Organic Molecules for Sustainable High Capacity Battery Application. *J. Mater. Chem. A* **2017**, *5*, 4430–4454. DOI: 10.1039/C6TA09760J.

4.3 Additives for Li-ion batteries

Targeted potential window in DMSO: 2.5 V and 3.5 V *vs*. Li^0/Li^+, converted to 3.9 to 4.9 V in absolute values. SAscore threshold for EvoMol = 3.0, filtering criteria – SAscore ≤ 3.

Table S7. EvoMol-generated candidate molecules, which converged in DFT-optimisation and possess DFT-computed reduction potentials in the target window.

SMILES	SAscore	Reduction potential, eV	
		MPNN	DFT
N=C1N=CC(=O)C2CCC(Cl)CC21	2.85	4.28	3.92
O=C1C(Br)=NC(=O)C2C(O)CCCC21	2.83	4.25	3.99
N=C1N=C(Cl)C(=O)C2CCCCC21	2.71	4.31	4.03
O=C1C=NC(=O)C2CCC(CO)CC21	2.63	4.20	4.08
O=C1C=NC(=O)C2CC(CO)CCC21	2.62	4.21	4.09
O=C1C=NC(=O)C2CCC(Cl)CC21	2.52	4.26	4.11
O=C1N=CC(=O)C2C(Cl)CCCC21	2.63	4.27	4.13
O=C1C=NC(=O)C2CC(CO)C(Br)CC21	2.98	4.24	4.14
O=C1C=NC(=O)C2CC(Cl)CCC21	2.52	4.26	4.16
CCC1CCCC2C1C(=O)C(Br)=NC2=O	2.77	4.20	4.18
O=C1N=CC(=O)C2C(Br)CC(Cl)CC21	2.95	4.29	4.18
O=C1C=NC(=O)C2CCC(Br)CC21	2.62	4.26	4.19
CC1CCCC2C1C(=O)C(Cl)=NC2=O	2.69	4.20	4.19
O=C1N=C(Br)C(=O)C2C(CO)CCCC21	2.88	4.23	4.19
O=C1N=CC(=O)C2C(F)CC(Cl)CC21	2.86	4.31	4.19
CC1CCC2C(C1)C(=O)N=C(Br)C2=O	2.66	4.19	4.22
O=C1C(Cl)=NC(=O)C2C(CO)CCCC21	2.80	4.26	4.22
O=C1C=NC(=O)C2CC(Cl)C(Br)CC21	2.93	4.28	4.22
O=C1N=C(Br)C(=O)C2CC(CO)CCC21	2.77	4.24	4.23
CC1CCC2C(C1Cl)C(=O)N=C(Br)C2=O	2.95	4.26	4.25
NCC1CCC2C(C1)C(=O)C(Br)=NC2=O	2.80	4.21	4.26
O=C1N=C(Br)C(=O)C2CCC(CF)CC21	2.97	4.23	4.27
O=C1N=C(Cl)C(=O)C2C(CBr)CCCC21	2.95	4.24	4.27
O=C1N=C(Cl)C(=O)C2C(Br)CCCC21	2.89	4.31	4.28
O=C1N=C(Br)C(=O)C2C1CCC(Cl)C2Cl	2.92	4.36	4.30
O=C1N=C(Br)C(=O)C2CCC(Cl)CC21	2.68	4.32	4.30
O=C1N=CC(=O)C2C(CO)CCCC21	2.78	4.19	4.30
O=C1N=C(Br)C(=O)C2CC(Cl)CCC21	2.68	4.31	4.31
O=C1N=CC(=O)C2C(O)CC(Br)CC21	2.96	4.23	4.32
O=C1N=C(Cl)C(=O)C2C(CCl)CCCC21	2.91	4.23	4.33
O=C1N=C(Cl)C(=O)C2CC(Br)CCC21	2.70	4.32	4.34
O=C1N=C(Br)C(=O)C2CC(Cl)C(Cl)CC21	2.86	4.36	4.35
O=C1N=C(Cl)C(=O)C2C(CO)CCCC21	2.81	4.25	4.35
N=C1N=C(Cl)C(=O)C2CC(Cl)CCC21	2.85	4.34	4.36
N=C1N=NC(=O)C2CCC(Cl)CC21	2.94	4.36	4.47
O=C1C=NC(=S)C2CCC(CO)CC21	2.98	4.33	4.49
O=C1N=C(Cl)C(=O)C2C(O)CCCC21	2.74	4.23	4.54

4.4 Anolytes for redox-flow batteries

Targeted potential window in water: -0.4V to 0.2V *vs*. SHE, converted to 4.04 eV to 4.64 eV in absolute values. SAscore threshold for EvoMol = 3.9, filtering criteria – SAscore ≤ 3.

Table S8. EvoMol-generated candidate molecules, which converged in DFT-optimisation and possess DFT-computed reduction potentials in the target window.

SMILES	SAscore	Reduction potential, eV	
		MPNN	DFT
CC1C(CO)CC2C(C1S(=O)(=O)F)C(=O)C1CC(Br)CC(Cl)C1C2=O	2.99	4.33	4.11
CS(=O)(=O)C1C(Cl)C(OOO)CC2C1C(=O)C1CC(Br)CCC1C2=O	2.97	4.33	4.11
CS(=O)(=O)C1C(Cl)C(COCBr)CC2C1C(=O)C1CC(Cl)CCC1C2=O	2.99	4.34	4.11
CCS(=O)(=O)C1CCC2C(C1)C(=O)C1CCC(CS(=O)O)CC1C2=O	2.98	4.34	4.14
CS(=O)(=O)C1C(Cl)C(COO)CC2C1C(=O)C1CC(Br)CCC1C2=O	2.90	4.35	4.14
O=C1C2CC(S(=O)(=O)O)CCC2C(=O)C2C1CCC(SO)C2O	2.76	4.33	4.14
O=C1C2CC(CO)C(O)C(O)C2C(=O)C2C1CCC(S(=O)(=O)O)C2Cl	2.85	4.32	4.15
COC1C(Cl)C(CBr)CC2C1C(=O)C1CC(S(=O)(=O)Br)CCC1C2=O	2.97	4.34	4.16
NCCC1CC(F)C2C(C1)C(=O)C1NC(Br)CCC1C2=O	2.71	4.34	4.22
O=C1C=CC(=O)C(O)=C1	2.47	4.25	4.36
